# A mosaic of colors: The influence of biotic and abiotic factors shaping flower color diversity across a tropical mountain ecosystem

**DOI:** 10.1002/ajb2.70147

**Published:** 2026-01-09

**Authors:** Maria Gabriela Gutierrez Camargo, Montserrat Arista, Pedro Joaquim Bergamo, Beatriz Lopes Monteiro, Leonor Patrícia C. Morellato

**Affiliations:** ^1^ Center for Research on Biodiversity Dynamics and Climate Change and Department of Biodiversity, Phenology Lab UNESP ‐ São Paulo State University, Biosciences Institute São Paulo Rio Claro Brazil; ^2^ Departamento de Biología Vegetal y Ecología, Facultad de Biología Universidad de Sevilla Seville Spain

**Keywords:** bee, campo rupestre, color contrast, color reflectance, color vision, environmental filters, hummingbird, plant community, pollination, UV patterns

## Abstract

**Premise:**

Flower color diversity within communities is shaped by biotic and abiotic factors. Pollinators often prefer specific colors, and floral pigments also help protect against abiotic factors such as ultraviolet (UV) radiation, precipitation, and temperature. Along altitudinal gradients, variations in biotic and/or abiotic conditions can drive the spatial distribution of flower color diversity at the community level.

**Methods:**

Across five vegetation types in the Brazilian campo rupestre, a highly diverse tropical mountain grassland with an environmental mosaic of vegetation types, we surveyed floral color traits of 179 plant species from 180 plots distributed along an altitudinal gradient (808–1427 m). We related flower color traits to pollination systems, abiotic factors (soil type, temperature, and precipitation), and elevation to investigate their influence on flower color diversity.

**Results:**

An association between flower colors and pollination systems was coupled with a functional divergence of color traits along the environmental mosaic, indicating that both, biotic and abiotic factors, shape color diversity in the campo rupestre. Despite this functional divergence, flower color diversity levels were similar across vegetation types and decreased slightly with elevation. Such maintenance of functional diversity contrasts with the sharp reductions in color diversity observed with elevation in temperate mountains.

**Conclusions:**

Our results indicate that flower color diversity is maintained across environmental gradients when pollination systems are unconstrained by elevation, a characteristic of old tropical mountain systems.

Flower color is a crucial communication channel between plants and pollinators, varying across individuals, species, and within communities (Camargo et al., 2019; Narbona et al., 2021). Different biotic and abiotic factors affect flower color distribution and diversity within a community (Arista et al.; 2013, Koski and Ashman, 2015; Bergamo et al., 2018; Gray et al., 2018; Shrestha et al., 2024). Pollinators’ color vision systems and floral preferences are key selective pressures for color diversity (Shrestha et al., 2013, 2014; Lunau et al., 2017; Camargo et al., 2019). Abiotic factors such as temperature, UV radiation, wind, and humidity can also alter flower color distribution (Devoto et al., 2005; Arista et al., 2013; Koski and Ashman, 2016; Peters et al., 2016; Perillo et al., 2017; Lefebvre et al., 2018; Monteiro et al., 2021; Shrestha et al., 2024). Furthermore, flower color distribution is directly linked to changes in plant abundance and flowering phenology (Martins et al., 2021b; Camargo et al., 2023). However, the extent that pollinators and the environment have independently influenced flower color diversity remains unclear.

Pollinator composition and plant–pollinator interactions often vary in heterogeneous habitats and along environmental gradients, making it challenging to analyze biotic and abiotic factors separately (Trøjelsgaard and Olesen, 2016). Pollinator composition and abiotic factors such as temperature change along altitudinal gradients (Perillo et al., 2017; Laiolo et al., 2018; Lefebvre et al., 2018; Monteiro et al., 2021; Dellinger et al., 2023). In tropical regions, bee abundance tends to decrease with increasing elevation, while hummingbird and dipteran abundance either increases or remains unchanged (Cuartas‐Hernández and Medel, 2015; Monteiro et al., 2021; Dellinger et al., 2023). This shift in pollinator composition can lead to altitudinal variation in flower color distribution due to selective pressures from different functional groups (Shrestha et al., 2014, 2024). Additionally, at high elevations, where UV light incidence increases and temperature decreases, the brightness of flowers may be lower due to higher concentrations of pigments such as anthocyanins, which offer protection against stressful conditions (Berardi et al., 2016; Gray et al., 2018). We also expect larger UV‐absorbing areas in petals of flowers at higher elevations, decreasing overall UV reflectance to protect reproductive parts from UV light (Koski and Ashman, 2015, 2016; Bergamo et al., 2018; Gray et al., 2018). Therefore, selective pressures from pollinators and abiotic drivers, alongside potential phylogenetic constraints on flower color, should shape the spatial patterns of flower color diversity along altitudinal gradients (Shrestha et al., 2014; Koski and Ashman, 2016).

Therefore, theory predicts lower flower color diversity at higher elevations compared to lower elevations, primarily due to increasing pressure from abiotic conditions. Pollinator diversity and abundance are also expected to decline with increasing elevation (Arroyo et al., 1982; Lara‐Romero et al., 2019). Reduced flower color diversity at high elevations may also reflect facilitative interactions, where functional convergence benefits plant species sharing pollinators in such harsh, pollinator‐depauperate environments (Bergamo et al., 2020). Conversely, high flower color diversity is often linked to functional divergence, resulting from plant species competing for pollinators at lower elevations (Van der Kooi et al., 2016; Bergamo et al., 2018). Considering the organization of plant species diversity within a community, especially in heterogeneous and highly diverse environments, is crucial, even though it is not always incorporated into analyses of flower color diversity.

The campo rupestre is a highly diverse, tropical, old‐growth vegetational mosaic, dominating the mountaintops of Brazilian Espinhaço Mountain range (Silveira et al., 2016; Morellato and Silveira, 2018). Plant diversity within this system is structured into distinct vegetation types dominated by a grassland matrix, primarily influenced by soil conditions across the altitudinal gradient (Rocha et al., 2016; Silveira et al., 2016; Mattos et al., 2019; Loiola et al., 2023). Such an environmental mosaic is significant because soil, in conjunction with elevation, also affects flower color and can potentially determine its distribution (Mtileni et al., 2024). In the heterogeneous landscape of the campo rupestre, plant populations can be spatially restricted to small areas, and seed dispersal is often limited by autochory (Guerra et al., 2016; Arruda et al., 2021). Consequently, pollinators, especially those involved in long‐distance pollination systems such as large bees and hummingbirds, are vital for landscape connectivity, promoting cross pollination and genetic diversity among areas (Monteiro et al., 2021). This role is evident in the spatial distribution patterns of plant species and flower color, as observed in closely related bee‐pollinated species that share similar flower colors in the campo rupestre (Camargo et al., 2023). For animal‐pollinated flowers, bee pollination predominates in the campo rupestre, followed by hummingbirds and other insects (Carstensen et al., 2014, 2016; Monteiro et al., 2021). However, previous studies in campo rupestre areas have reported differences in pollinator diversity along altitudinal gradients and across vegetation types (Rodrigues and Rodrigues, 2015; Fernandes et al., 2016; Perillo et al., 2017; Monteiro et al., 2021) and in the influence of pollinator visual systems on flower color diversity (Camargo et al., 2019; 2023).

In this study, we used pollinator visual models to analyze flower color diversity across an environmental mosaic of vegetation types and elevations within a campo rupestre area. We tested (1) whether flower color is associated with pollination systems, (2) whether flower color exhibits functional convergence or divergence (i.e., similarity or dissimilarity among co‐occurring plant species) across environmental variables, based on the visual systems of bees and hummingbirds, and (3) whether flower color diversity (i.e., composition of flower colors) varies between elevations and vegetation types. Flower color was represented by both categorical and quantitative color variables, with the quantitative ones calculated according to the perceptual models of the two most abundant pollinator groups in the area: bees and hummingbirds (Camargo et al., 2019; Monteiro et al., 2021).

We expected to find differences in the distribution of flower colors related to pollination systems, vegetation types, and elevation. Specifically, we anticipated the number of UV‐reflecting flowers to increase with elevation due to higher radiation levels (Fernandes et al., 2016; Koski and Ashman, 2016). Considering the intensification of environmental filters at higher elevations for both pollinators and plants, we expected a clustered distribution of flower colors and functional convergence, leading to reduced flower color diversity along the elevation.

## MATERIALS AND METHODS

### Study area and species survey

The study area is located within the Serra do Cipó National Park and its buffer zone (Environmental Protected Area Morro da Pedreira) at the southern end of the Espinhaço Mountain range (Cadeia do Espinhaço), which includes the municipalities of Santana do Riacho and Jaboticatubas, Minas Gerais State, Brazil, hereafter referred to as Serra do Cipó (Appendix S1: Figure S1). The climate is seasonal, characterized by dry winters (May to September) and wet summers (October to April), with a mean annual rainfall of 1500 mm (Fernandes et al., 2016). An altitudinal gradient of abiotic conditions exists across Serra do Cipó, ranging from 824 to 1400 m a.s.l., with temperature decreasing and air humidity and wind speed increasing with elevation (Fernandes et al., 2016). The typical campo rupestre (Silveira et al., 2019) dominates areas above 1100 m a.s.l., though patches of campo rupestre can occur from approximately 900 m a.s.l. (Mattos et al., 2019). The vegetation mosaic comprises five distinct vegetation types: rocky outcrops covered by shrubs, small trees, and herbs, surrounded by a matrix of sandy, stony, or wet grasslands dominated by Poaceae and Cyperaceae; other herbs such as Asteraceae, Xyridaceae, and Velloziaceae; and sparse, small shrubs (Mattos et al., 2019; Loiola et al., 2023). At elevations below 900 m, cerrado savanna physiognomies predominate, with small trees and shrubs, primarily Fabaceae, Asteraceae, and Malpighiaceae species, immersed in a continuous herbaceous layer mainly composed of Poaceae and Cyperaceae species (Mattos et al., 2019; Loiola et al., 2023).

We analyzed the flower color of 179 species, based on human and pollinator color vision (detailed below). The species were systematically surveyed (detailed by Mattos et al., 2019) across 180 permanent plots (1 m^2^) distributed among five sites at elevations from 808 to 1427 m in the study area (Appendix S1: Figure S1C, Table S1). At each elevation, 36 plots were distributed along four 250‐m transects (see Mattos et al., 2019; Loiola et al., 2023). The plots encompassed various vegetation types, with typical cerrado vegetation below 900 m elevation and campo rupestre above 900 m, further subdivided into rocky outcrops, sandy grasslands, stony grasslands, and wet grasslands (Appendix S1: Figure S1). Therefore, we evaluated color trait diversity of species in five vegetation types across five elevations (Appendix S1: Figure S1).

At the site around 824 m (locally called Rio Cipó), open and typical cerrado vegetation predominates, with few rocky outcrops. The site around 1101 m (Cedro) is primarily campo rupestre, consisting mainly of sandy grasslands, rocky outcrops, and wet grasslands, with some cerrado scrubland patches. The site at 1255 m (Pedra do Elefante) is exclusively campo rupestre, characterized by stony grasslands and rocky outcrops, with some sandy and wet grasslands. The site at 1303 m (Quadrante 16) also presents only campo rupestre, predominantly sandy and stony grasslands, with some rocky outcrops and wet grasslands. The fifth site at 1420 m (Alto Palácio) is dominated by sandy and stony grasslands, with few rocky outcrops. Each study site was described in detail by Mattos et al. (2019). We recorded species occurrences in each plot to obtain species frequencies per elevation and vegetation type.

We identified most plant species when collecting flower color data, based on previous studies of the same sites (Rocha et al., 2016; Mattos et al., 2019, 2021; Loiola et al., 2023) and on specialized bibliography. Voucher specimens are deposited in the Herbarium Rioclarense collection (HRCB) of the São Paulo State University (UNESP), Rio Claro, São Paulo State, Brazil.

For environmental data, we used mean annual temperature (°C), relative humidity (%), wind speed (m/s), and solar radiation (W/m^2^), collected from Onset climatic stations at each of the five sites (PELD‐CRSC project; Fernandes et al., 2016). Although elevation was recorded for each of the 180 plots, elevation was analyzed by transect. Differences in the scales of assessing environmental variables were accounted for in the statistical analyses. We included five environmental variables: elevation, wind speed, humidity, solar radiation, and temperature because they could influence pollinator distribution and flower color.

### Pollination systems

We determined the main pollinator vectors for each species (pollination systems) using a comprehensive database of plant–pollinator interactions from previous studies in the same area. The database was compiled from direct observations and systematic bibliographic survey (Monteiro et al., 2021, 2025). The primary pollinator for each plant species was defined as the one with the most recorded interactions (Monteiro et al., 2021). For this study, we combined the pollination systems into three groups: bees, hummingbirds, and other systems. Other systems encompassed the less‐frequent pollinator systems such as bats, wasps, flies, moths, and butterflies (Monteiro et al., 2021).

### Color reflectance and color variables

We measured flower color reflectance with a spectrometer (Jaz Modular Optical Sensing Suite; Ocean Optics, Orlando, FL, USA‐) sensitive to wavelengths from 200 to 1100 nm. For each species, flower color was represented by the mean reflectance of 10 to 20 flowers. We measured the flower periphery, which in general corresponded to the petals, collected from different individuals (Dalrymple et al., 2015; Camargo et al., 2019). We also measured reflectance of internal floral structures (e.g., floral guides, exposed anthers, or stigmas) and captured UV photos to detect UV reflection and classify the presence of UV and color patterns of 10 flowers per species, defined below. Additionally, we measured the reflectance of 10 leaves collected from each species to calculate the mean reflectance of the flowers background (Martins et al., 2021a). All flowers and leaves were kept wet in humid chambers before reflectance measurements.

**Figure 1:**
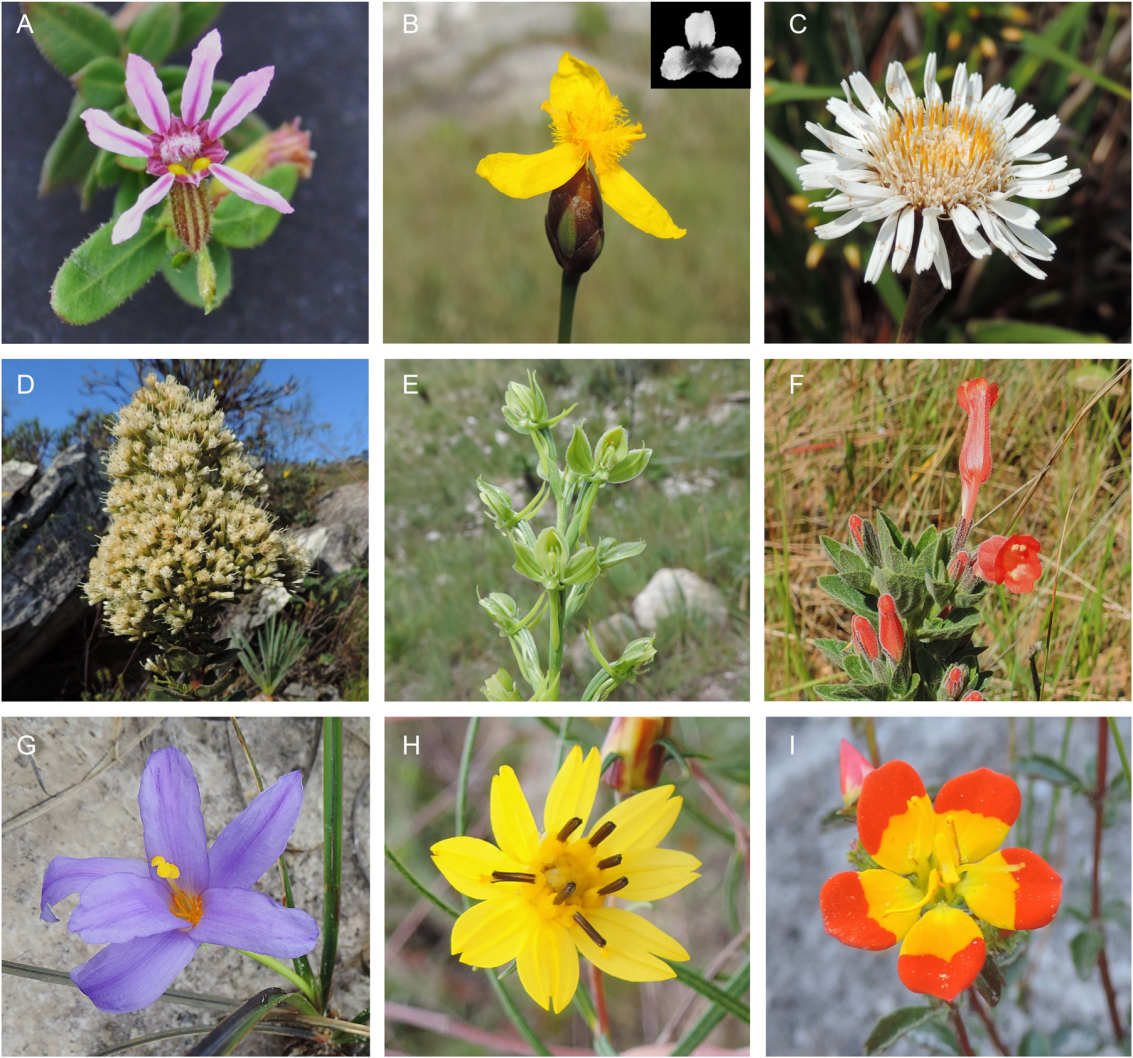
Examples of flowers of different human‐ and bee‐color categories (in parentheses) sampled in the studied campo rupestre. (A) Pink/purple (bee‐uvblue) bee‐pollinated flower of *Cuphea cf. linarioides* with floral guides (dark pink lines in the petals) and pollen/anther mimicry (yellow UV‐absorbing spots in the tube entrance); (B) bee‐pollinated flower of *Xyris trachyphylla* with UV‐pattern (UV‐photo in detail) composed of yellow UV‐reflecting petals (bee‐uvgreen) and yellow UV‐absorbing center, which also presents pollen/anther mimicry (yellow UV‐absorbing staminodes hairs); (C) white (bee‐bluegreen) bee‐pollinated flowers of *Richterago polymorpha*; (D) whitish (bee‐bluegreen) flowers of *Mikania sessilifolia* and (E) the green (bee‐bluegreen) flowers of *Habenaria magniscutata* both pollinated by pollinators other than bees and hummingbirds; (F) red (bee‐uvblue) hummingbird‐pollinated flowers of *Ruellia densa*; (G) pink/purple (bee‐blue) bee‐pollinated flower of *Vellozia albiflora* with pollen/anther mimicry (yellow UV‐absorbing stigma); (H) yellow (bee‐green) bee‐pollinated flowers of *Aspilia jolyana* with pollen/anther mimicry (petals of disk flowers); (I) bee‐pollinated flower *Cambessedesia semidecandra* with UV pattern (UV photo in detail) composed of the red (bee‐uv) flower periphery and yellow UV‐absorbing center, which also mimics the pollen/anther color.

For categorical color traits, we classified flower colors at the species level based on (1) human color vision (pink/purple, white, yellow, red, whitish, and green) and additional information on UV reflectance or absorbance and on (2) bee color vision (blue, blue‐green, UV, UV‐blue, green, UV‐green) (Chittka et al., 1994). We also noted (3) color patterns, defined as two or more colors within the floral display (Lunau et al., 1996, 2017). Furthermore, we assessed whether these color patterns were represented by (4) floral guides, (5) pollen/anther mimicry (structures exhibiting the same color as pollen and anthers, generally yellow UV‐absorbing; Lunau et al., 2017, 2024), and (6) UV‐patterns (combinations of UV‐reflecting and UV‐absorbing colors; Figure 1). Color patterns related to floral guides, exposed anthers, pollen/anther mimicking structures, and UV‐patterns influence flower attraction and discrimination, thereby contributing to flower color diversity. These visual traits are linked to the preferences of different pollinator groups and abiotic factors such as UV‐radiation exposure (Hempel de Ibarra et al., 2015; Koski and Ashman, 2016; Papiorek et al., 2016; Camargo et al., 2019; Koski et al., 2020).

Additionally, we obtained continuous color traits by analyzing the distribution of flower color (color loci) in bee and hummingbird perceptual color spaces. We focused on these color systems because bees and hummingbirds are the predominant pollinators at the study area (Carstensen et al., 2014, 2016; Camargo et al., 2019; Monteiro et al., 2021). The color space for bee trichromatic color vision is represented by a hexagon (Menzel and Backhaus, 1991; Chittka, 1992; Lunau et al., 2011); bird tetrachromatic color vision is represented by a tetrahedron (Endler and Mielke, 2005). The vertices of these diagrams correspond to the sensitivity of each photoreceptor: UV, blue (s), and green (m) wavelengths for bees and ultraviolet (UV), blue (s), green (m), and red (l) wavelengths for birds. Color loci positions correspond to coordinates calculated based on the stimulus from flower reflectance, specifically the quantum catches captured by each photoreceptor (Chittka, 1992; Endler and Mielke, 2005). We used the photoreceptor sensitivities of the *Bombus terrestris* visual system (Peitsch et al., 1992) and the *Leiothrix lutea* UVS visual system (Vorobyev et al., 1998) to calculate bee and bird quantum catches, respectively. In model calculations, we used the mean reflectance spectra of leaves collected for all species as the leaf‐green background (Camargo et al., 2014) and the D65 standard daylight as the ambient light (Wyszecki and Stiles, 1982).

Based on bee color vision, we calculated dominant wavelength, spectral purity, and brightness (Lunau et al., 1996; Rohde et al., 2013) and chromatic and green contrasts between the predominant flower color and the leaf background (Chittka, 1992) and quantum catches for UV (s), blue (m), and green (l) photoreceptors. Based on bird color vision, we calculated color spectral purity (Stoddard and Prum, 2011), chromatic and achromatic contrasts between the flower color and the leaf background (according to Vorobyev and Osorio, 1998), and UV (uv), blue (s), green (m), and red (l) photoreceptor quantum catches. For detailed formulas of color variables, refer to Camargo et al. (2019). We utilized color variables previously described as the primary spectral properties and important floral signals for bees and birds (Lunau et al., 1996, 2007; Spaethe et al., 2001; Cazetta et al., 2009; Dyer et al., 2016; Camargo et al., 2019; Van der Kooi et al., 2021).

### Statistical analyses

To assess flower color distribution in the campo rupestre, we tested its variation in pollination systems (bees, hummingbirds, and other systems), elevation (sites at 824, 1101, 1255, 1303, and 1421 m a.s.l.), environmental variables (mean annual temperature, relative air humidity, wind speed, and solar radiation), and among vegetation types (cerrado, rocky outcrop, sandy grassland, stony grassland, and wet grassland) using both categorical and quantitative color traits derived from flower reflectance spectra. The data set used for all analyses is available at Zenodo repository (https://doi.org/10.5281/zenodo.17237031).

#### Flower color and pollination systems

To address our first question regarding the relationship between flower color traits and pollination systems, we analyzed species occurrences and their corresponding categorical color variables and whether there was a relationship with the frequency of each pollination system category using a canonical correspondence analysis (CCA). We restricted this analysis to categorical variables to avoid circularity that would arise from using continuous traits derived from pollinator visual models in conjunction with the corresponding pollinator systems (bee and hummingbird).

#### Color trait convergence and divergence

To address our second question, which explored potential environmental filters constraining the diversity of color traits, we followed the RLQ framework proposed by Pillar et al. (2009) and Pillar and Duarte (2010), building four matrices using (1) W, representing the occurrence of species in each community (i.e., each plot); (2) B, containing the set of color traits describing each species, which included quantitative bee‐ and bird‐color traits calculated per species; (3) E, representing the ecological gradient using environmental variables, including elevation, vegetation type, temperature, relative air humidity, wind speed, and radiation; and (4) P, a phylogenetic matrix with distances between pairs of species. We applied standardization methods (centering and scaling data between 0 and 1 for all variables) and removed plots with missing values to properly implement the RLQ framework. This framework is designed to analyze environmental filters on ecological communities by relating combinations of functional traits to combinations of environmental variables (Dolédec et al., 1996).

To assemble the phylogenetic matrix (P), we used the angiosperm megaphylogeny proposed by Zanne et al. (2014). This phylogenetic tree, including estimated branch lengths, is available via the function S.PhyloMaker in R version 4.0.0 (R Core Team, 2020) and the megaphylogeny PhytoPhylo of Qian and Jin (2016) and their third scenario. To test for phylogenetic signals in the quantitative color variables, we used Blomberg's *K* (Blomberg et al., 2003). When significant (*P* < 0.05), *K* values around 1 indicate no phylogenetic signal, *K* values higher than 1 indicate a positive signal, and *K* values lower than 1 indicate a negative signal (Ackerly, 2009).

We used three versions of the B matrix for the analyses: (1) the entire color space, incorporating bee‐ and bird‐color traits; (2) only bee‐color traits (including only bee‐pollinated plants); and (3) only bird‐color traits (restricted to hummingbird‐pollinated plants). These matrices were constructed using Gower distances among all color traits or among only bee‐color traits or only bird‐color traits. We tested for both trait‐convergence and trait‐divergence assembly patterns, generating *P*‐values through permutation testing based on null models (Pillar et al., 2009; Pillar and Duarte, 2010). Trait convergence suggests environmental filtering and facilitation, while divergence is more indicative of competition (Pillar et al., 2009; Camargo et al., 2023). Additionally, we applied a trait‐selection procedure to further investigate how trait‐convergence or trait‐divergence assembly patterns relate to elevation. In this context, the RLQ framework identifies which traits contributed most to convergence or divergence patterns by applying multiple stepwise linear models for each trait. These linear models included the community weighted means (CWM) of color traits (one per model), with elevation as a predictor. We used linear regression plots to visualize the relationships between the color traits and the traits selected by the RLQ framework.

#### Flower color diversity in the environmental context

To address our third question, we first calculated the proportion of pollination systems (bee, hummingbird, and other), human color categories (with UV), bee color categories, and color patterns along both the elevational gradient and across vegetation types. The proportion of pollination systems and color categories provided an initial characterization of the main patterns of variation in color traits within the environmental context. For a preliminary description of color diversity, we calculated the distances among flower color loci for bee‐pollinated flowers within the bee hexagon and for bird‐pollinated flowers within the bird tetrahedron. We considered discrimination thresholds of 0.2 hexagon units (HU) for bees and 0.1 just noticeable differences (JND) for birds (Vorobyev et al., 1998; Martins et al., 2021b). Subsequently, we calculated the mean distance among flower color loci (for both bee‐ and hummingbird‐pollinated plants) found at each elevation and in each vegetation type (Shrestha et al., 2014).

To analyze flower color diversity (i.e., the composition of flower colors considering all traits), we calculated the standardized effect sizes of mean pairwise distances (SES MPD) by shuffling communities while maintaining species occurrence frequency and sample richness after 999 randomizations based on the Gower distance among all color traits (Webb et al., 2002; Swenson, 2014). The SES MPD is a pairwise metric that captures the overall diversity of species in a sample; however, it is not particularly recommended for detecting fine‐scale patterns (Swenson, 2014). We calculated the SES MPD for all color traits, for bee‐pollinated plants in the bee color space, and for hummingbird‐pollinated plants in the hummingbird color space. To further investigate trait diversity in communities, we also calculated the standardized effect sizes of mean nearest neighbor distances in a plot (SES MNTD) to determine whether species were clustered or dispersed in color space within a plot (Webb et al., 2002; Swenson, 2014). We compared the SES MPD and SES MNTD results, and because there was no divergence between them, we present only the results for SES MPD. Finally, we used the *z*‐scores for the SES MPD in linear mixed model**s** to test for differences between vegetation types and along the elevational gradient, with transects included as a random factor (Zuur et al., 2007). We did not conduct a linear mixed model for SES MPD of hummingbird‐pollinated plants because these plants were almost entirely absent from vegetation types other than rocky outcrops. We plotted the relationship between SES MPD and elevation separately for each vegetation type to visually inspect potential differences in flower color diversity across the environmental context.

We carried out all the analyses in R version 4.0.0 (R Core Team, 2020), utilizing the R package pavo (Maia et al., 2013) for calculating color variables, the R package phytools (Revell, 2012) for constructing the phylogenetic tree, the picante package for phylogenetic analysis (Kembel et al., 2010), and the SYNCSA package (Pillar, 2012) for functional and phylogenetic trait analysis.

## RESULTS

### Flower color and pollination systems

Of the 179 species analyzed (Appendix S1: Table S1), 60% were animal‐pollinated plants, which were surveyed by Monteiro et al. (2021) at the same sites, with a similar proportion of plants per pollination system: 75% primarily pollinated by bees (134 species), 8% by hummingbirds (14 species), and 17% by other systems (31 species). The other systems category predominantly comprised diverse insects (17 species), followed by flies (6 species), moths and wasps (3 species each), and butterflies and bats (1 species each) (Appendix S1: Table S1).

Considering human color categories associated with UV reflectance/absorbance, pink UV‐absorbing (28%), white UV‐absorbing (18%), and yellow UV‐reflecting (17%) flowers were most prevalent among species (Appendix S1: Table S2). According to categories in bee vision (i.e., color loci position in the bee‐hexagon), most flowers were blue‐green (34%), blue (29%), and UV‐green (17%) (Appendix S1: Table S2). Color patterns were present in 66% of species; of these, 23% displayed UV‐patterns, 32% had pollen or anther mimic structures, and 13% exhibited other floral guide types (Appendix S1: Table S2).

Correspondence analyses, based on the frequency of pollination systems, revealed significant correlations between flower colors and pollination systems (Appendix S1: Figure S2, Table S2) (*P* < 0.001, accounting for 68.6% of the variability). Regarding human color categories associated with UV reflection/absorbance, bee‐pollination correlated with UV‐absorbing and UV‐reflecting pink and yellow flowers. Hummingbird pollination was linked to red UV‐absorbing flowers, while other systems were primarily associated with white UV‐reflecting and whitish UV‐absorbing flowers (Appendix S1: Figure S2A). White UV‐absorbing flowers were equally distributed among pollination systems (Appendix S1: Figure S2A, Table S2). In terms of bee vision, bee‐pollinated flowers corresponded to blue and UV‐green colors, hummingbird‐pollinated flowers to UV‐blue and green, and other systems to blue‐green (Appendix S1: Figure S2B).

Approximately 80% of bee‐pollinated and 60% of hummingbird‐pollinated flowers displayed color patterns, whereas flowers with no color pattern predominated among those pollinated by other systems (84%, *P* < 0.001, *G* = 87.49). Of the 134 bee‐pollinated species, 30% presented a UV‐pattern, and 42% had pollen/anther mimicking structures (Appendix S1: Table S2).

### Color trait convergence and divergence

The RLQ analyses revealed overall functional divergence in color traits related to the environmental context, indicating dispersion in color space among plants co‐occurring in plots (Table 1). Although phylogenetic structure was significantly related to environmental variables, no phylogenetic signal was detected at the community level (Table 1). When analyzing the color trait space separately for bee‐ and bird‐color traits, we observed that the patterns shifted to convergence and divergence for bee vision and to null‐patterns for hummingbird vision (Table 1).

**Table 1:** Results of permutation tests to assess the significance of (1) trait convergence, (2) trait divergence, (3) phylogenetic signal related to environmental variables, (4) phylogenetic structure related to trait convergence, (5) phylogenetic structure related to trait divergence, (6) removing the phylogenetic structure related to trait convergence, and (7) removing the phylogenetic structure related to trait divergence within the campo rupestre (Serra do Cipó, Minas Gerais State, Brazil), after 1000 randomizations (Pillar et al., 2009; Pillar and Duarte, 2010). For these analyses, “trait” refers to functional diversity indices calculated using all continuous and categorical flower color traits, only bee functional traits, and only hummingbird functional traits. Significant values (*P* < 0.05) are in bold.

Functional pattern	All	Bees	Humm
(1) Trait convergence: assembly patterns	0.130	**0.016**	0.318
(2) Trait divergence: assembly patterns	**0.008**	**0.01**	0.351
(3) Phylogenetic structure: environment	**0.001**	**0.001**	**0.001**
(4) Phylogenetic structure: trait convergence	0.803	0.531	0.893
(5) Phylogenetic structure: trait divergence	0.236	0.503	0.746
(6) Removing phylogeny: trait convergence	0.23	0.079	0.379
(7) Removing phylogeny: trait divergence	**0.029**	**0.016**	0.364

No phylogenetic signal was observed at the species level, either when considering all color traits together or for bee‐color traits separate from bird‐color traits. Most color variables did not exhibit a phylogenetic signal (*P* > 0.05). However, blue quantum catches and chromatic contrasts for bees, and UV quantum catches for birds showed negative phylogenetic signals (*P* < 0.05, *K* < 1). These variables were phylogenetically structured, indicating phylogenetic dispersion among the studied species (Appendix S1: Table S3).

**Figure 2:**
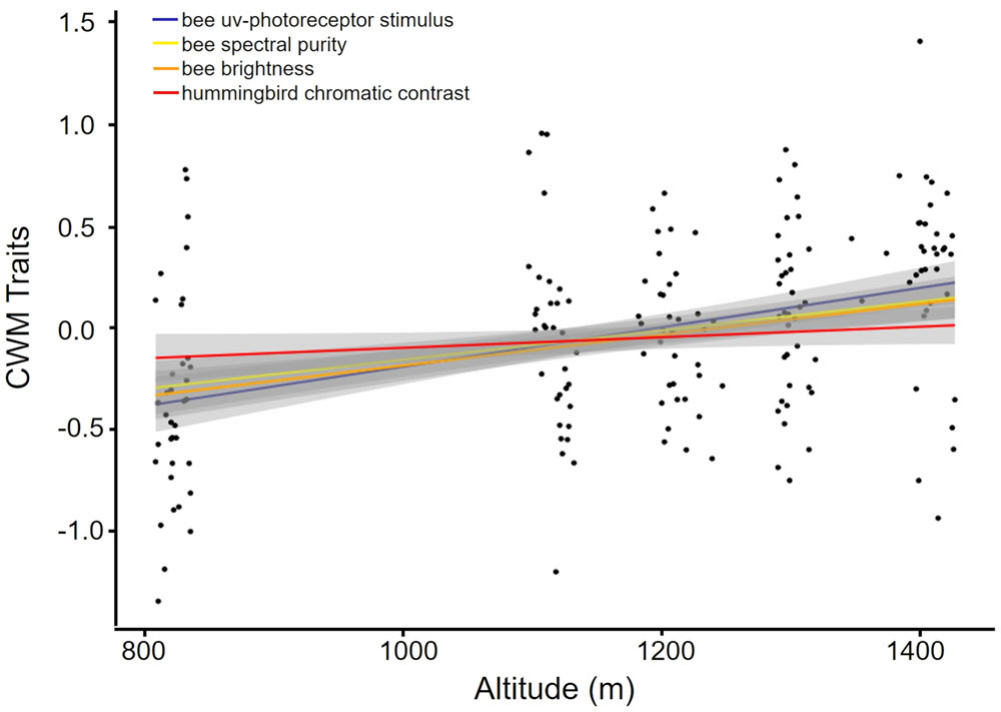
Linear regression between the community weighted mean (CWM) of selected flower color traits that have contributed most to divergence and clustering patterns in the campo rupestre (Serra do Cipó, Minas Gerais State, Brazil). The blue line represents the stimulus in the bee UV photoreceptor, yellow represents spectral purity according to bee color vision, orange represents brightness according to bee color vision, and red represents the chromatic contrast against the green background of leaves according to hummingbird color vision.

Four variables exhibited clustering patterns, as shown by the RLQ analysis: bee UV photoreceptor stimulation (s‐bee), bee spectral purity (S‐bee), bee brightness, and hummingbird chromatic contrast with the background (CCB‐hummingbird) (Figure 2, Table 2). The community weighted mean (CWM) of all these variables slightly increased with elevation (Figure 2).

**Table 2:** Results of the linear regression models of the relationship between color traits and elevation (one LM per color trait). Variables selected by the RLQ analysis are bolded, indicating these color traits exhibited clustered patterns and increased along the altitudinal gradient. CCB = chromatic contrast with the background; ACB = achromatic contrast with the background.

Variable	*P*	*R* ^2^	Direction/relationship
**Bee uv stimulus**	<0.001	0.162	+
Bee blue stimulus	0.004	0.045	+
Bee green stimulus	<0.001	0.102	+
**Bee brightness**	<0.001	0.135	+
**Bee spectral purity**	<0.001	0.156	+
Bee green contrast	0.03	0.02	+
**Hummingbird CCB**	<0.001	0.105	+
Hummingbird ACB	0.02	0.028	+
Hummingbird UV stimulus	0.07	0.018	NA
Hummingbird red stimulus	0.32	0.005	NA

### Flower color diversity in the environmental context

**Figure 3:**
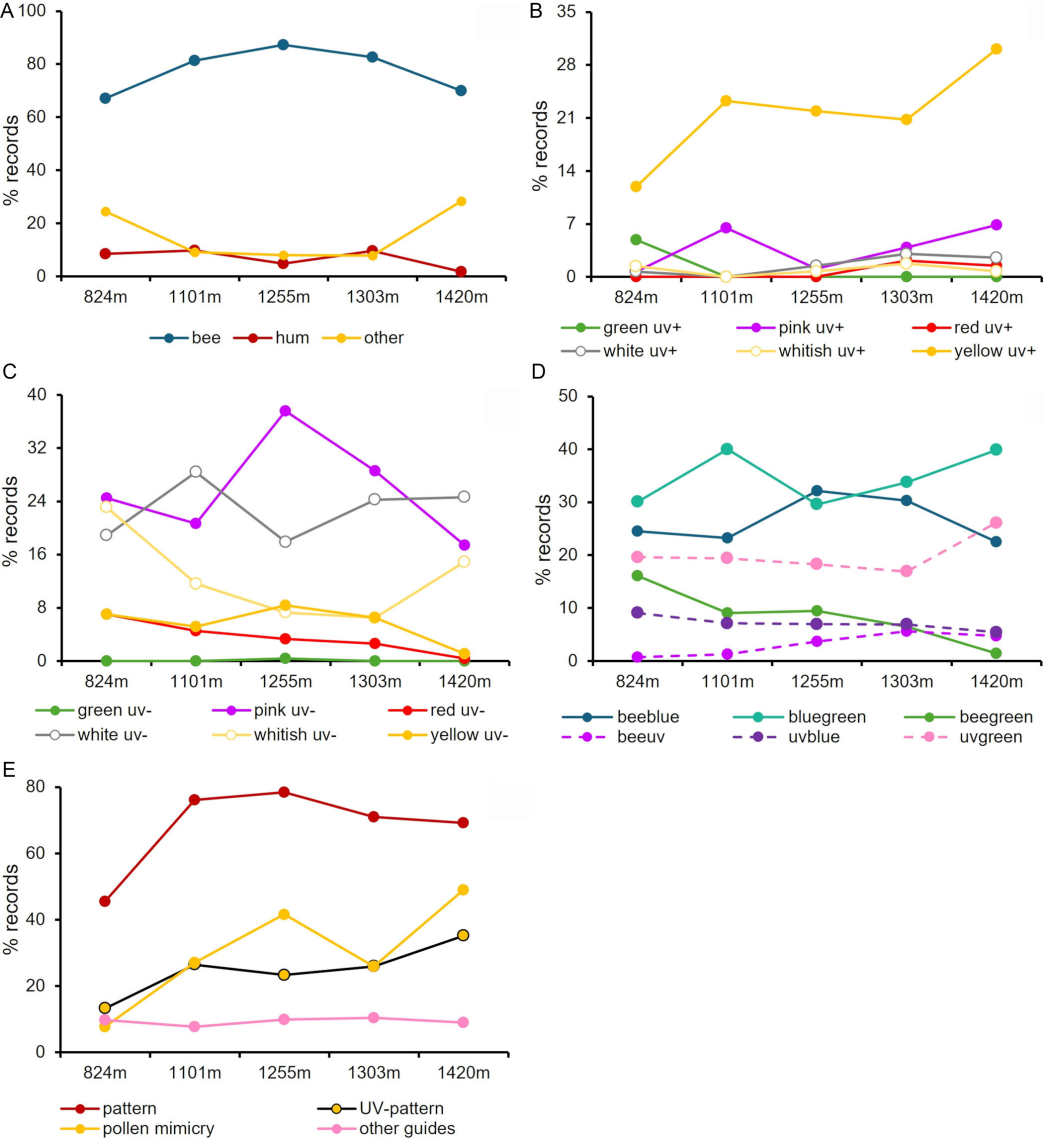
Frequency (% of records) per altitude of the three pollination system (A); human color categories associated with UV‐reflectance: (B) UV‐reflecting; (C) UV‐absorbing; (D) bee color categories according to the bee hexagon (Chittka et al., 1994), and (E) flowers exhibiting color pattern, pollen/anther mimicking structures, floral guides, and UV patterns at each of the five altitudes in the campo rupestre (Serra do Cipó, Minas Gerais State, Brazil). Across the 180 plots established and the five sites, we collected 1079 records of animal‐pollinated species with flower reflectance data (see Appendix S1 for detailed information).

**Figure 4:**
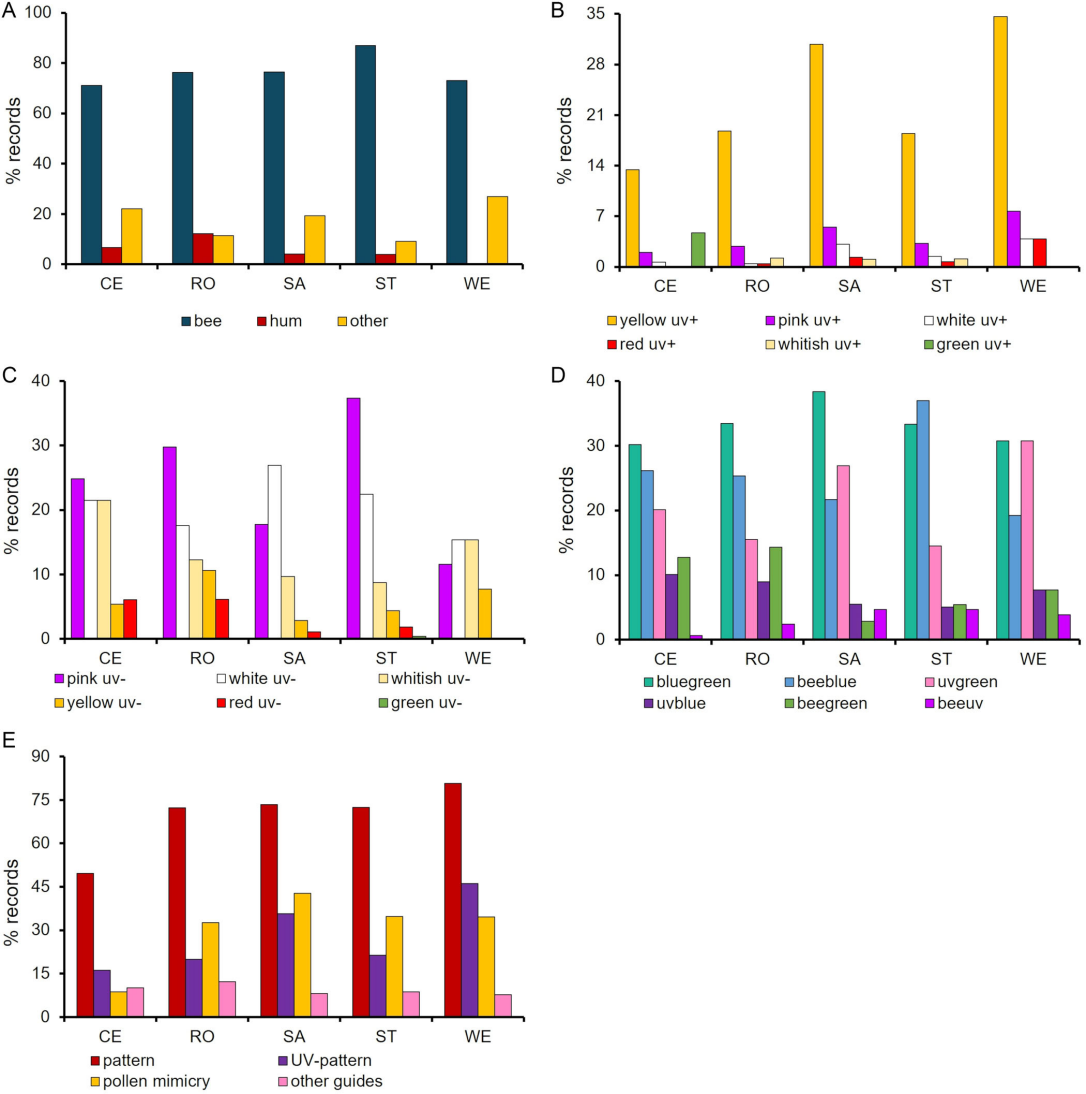
Frequency (% of records) by vegetation types of the three (A) pollination system, (B) human color categories associated with the: UV‐ reflecting and (C) UV‐absorbing flowers; (D) bee color categories according to the bee hexagon (Chittka et al., 1994); and (E) flowers presenting color pattern, pollen/anther mimicking structures, floral guide and UV‐pattern at each of the five vegetation types of the studied campo rupestre (Serra do Cipó, Minas Gerais State, Brazil). Across the 180 plots established in each study site, we collected a total of 1079 records of the animal‐pollinated species with flower reflectance data (see Appendix S1 for detailed information). CE, cerrado; RO, rocky outcrop; SA, sandy grassland; ST, stony grassland; WE, wet grassland.

Bee pollination was the most important pollination system, followed by other systems and hummingbirds, across all elevations (Figure 3; Appendix S1: Table S1) and vegetation types (Figure 4; Appendix S1: Table S1). Bee‐pollinated flowers were predominantly observed at intermediate elevations, while other systems showed an inverse pattern, increasing in both the lowest and highest elevations. The frequency of hummingbird‐pollinated flowers, conversely, decreased at the highest elevation (Figure 3A).

Yellow UV‐reflecting flowers increased with elevation (Figure 3B), whereas yellow and red UV‐absorbing flowers decreased (Figure 3C). Pink UV‐absorbing flowers predominated at intermediate elevations, and whitish UV‐absorbing flowers were more common at the extremes (lowest and highest elevations) (Figure 3C). Yellow UV‐reflecting flowers occurred across all vegetation types (Figure 4B). Pink UV‐absorbing flowers were more frequent in stony grasslands and rocky outcrops, white UV‐absorbing in sandy grasslands, and red UV‐absorbing flowers in rocky outcrops and cerrado (Figure 4C).

Most bee‐color categories decreased at the highest elevation, with only blue‐green and UV‐green increasing with elevation; blue flowers predominated at intermediate elevations (Figure 3D). Blue‐green flowers prevailed in almost all vegetation types (Figure 4D). Bee‐colors were similar between rocky outcrops and cerrado, with differences observed in sandy, stony, and wet grasslands, which showed higher frequencies of species with UV‐green and blue flowers, and similar frequencies of blue‐green and UV‐green flowers, respectively (Figure 4D). Flowers with color patterns were less common at the lowest elevation, specifically in cerrado vegetation, while they were more common at higher elevations in typical campo rupestre vegetation (Figures 3, 4). Flowers with pollen/anther mimicking structures and UV‐patterns were associated with typical campo rupestre vegetation types and increased with elevation (Figures 3E, 4E).

Lower values of mean distances between pairs of color loci, calculated according to bee and bird visual systems, were observed at both the lowest elevation (0.22 HU and 0.21 JND) and above 1000 m, with the highest values occurring at intermediate elevations (0.28 HU and 0.28 JND) (Appendix S1: Figure S3). Among the vegetation types, the lowest mean distance was observed in sandy grassland for hummingbird‐pollinated flowers and in cerrado for bee‐ pollinated flowers (0.17 JND and 0.23 HU) (Appendix S1: Figure S3). With the exception of the cerrado vegetation, all communities exhibited high proportions of flower color loci discriminable by bees (>0.2 HU) and birds (>0.1 JND), exceeding 70% for hummingbird‐pollinated flowers and around 60% for bee‐pollinated flowers (Appendix S1: Figure S3).

**Figure 5:**
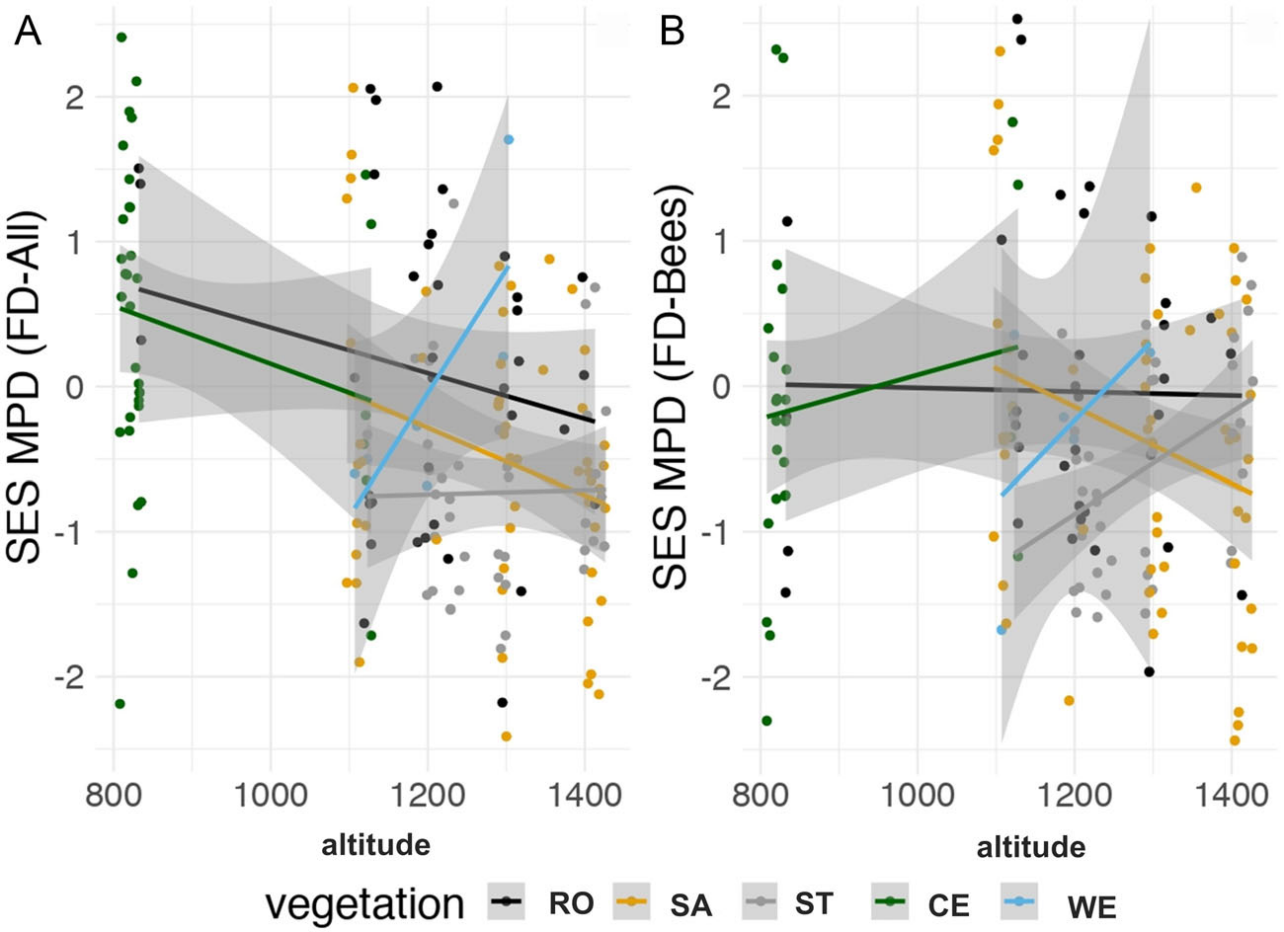
Relationship between flower color diversity and elevation across vegetation types in the campo rupestre. Flower color diversity was quantified as the standardized effect size of mean pairwise differences (SES MPD) of functional dispersion (Webb et al., 2002) calculated for (A) all color traits (FD‐All) and (B) only bee color traits of bee‐pollinated plants (FD‐Bee). CE, cerrado; RO, rocky outcrop; SA, sandy grassland; ST, stony grassland; WE, wet grassland.

Flower color diversity (MPD *z*‐scores considering all color traits) decreased with elevation, a pattern most evident in sandy grasslands (Figure 5A, Table 3). Nevertheless, most vegetation types showed no trend or even an increasing trend (as in wet grasslands) in flower color diversity with elevation (Figure 5A). For bee‐pollinated plants, flower color diversity did not change with elevation or between vegetation types (Figure 5B, Table 3).

**Table 3:** Results of the linear mixed models for the relationship between flower color diversity (MPD) and with vegetation type (one LMM per group of plants). “All” refers to MPD calculated using color traits from all species; “Bee” refers to MPD calculated using color traits solely from bee‐pollinated species. Vegetation types: CE, cerrado; SA, sandy grassland; ST, stony grassland; WE, wet grassland. Results shown are for comparison of each of these vegetation types to rocky outcrop (RO).

Model	Term	Estimate	Error	*t*	*P*	Marginal *R* ^2^	Conditional *R* ^2^
SES.MPD (All)	(Intercept)	2.39	0.72	3.31	1.00	0.21	0.22
SES.MPD (All)	Elevation	0	0	–3.03	0	0.21	0.22
SES.MPD (All)	SA	–0.52	0.25	–2.10	1.00	0.21	0.22
SES.MPD (All)	ST	–0.76	0.26	–2.93	1.00	0.21	0.22
SES.MPD (All)	CE	–0.48	0.32	–1.51	1.00	0.21	0.22
SES.MPD (All)	WE	–0.24	0.42	–0.56	1.00	0.21	0.22
SES.MPD (Bees)	(Intercept)	0.77	0.81	0.96	0.34	0.09	0.09
SES.MPD (Bees)	elevation	0	0	–1.18	0.24	0.09	0.09
SES.MPD (Bees)	SA	–0.28	0.23	–1.24	0.22	0.09	0.09
SES.MPD (Bees)	ST	–0.43	0.24	–1.78	0.08	0.09	0.09
SES.MPD (Bees)	CE	0.10	0.32	0.31	0.76	0.09	0.09
SES.MPD (Bees)	WE	–0.04	0.45	−0.08	0.94	0.09	0.09

## DISCUSSION

Here, we analyzed floral color traits and diversity along an elevational gradient encompassing a mosaic of vegetation types within the hyperdiverse tropical grassland known as the campo rupestre. Overall, we found a correspondence between floral colors and pollination systems, coupled with trends of functional divergence (i.e., a tendency for co‐occurring species to exhibit distinct floral colors). Nevertheless, floral color diversity remained high and similar across both elevation and vegetation types, with only a few exceptions. Such maintenance of high levels of color diversity suggests that color diversity in campo rupestre tropical grassland is not constrained by changing environmental conditions, a functional stability that contrasts with patterns observed in temperate and other tropical high elevational gradients (Bergamo et al., 2018; Gray et al., 2018; Tai et al., 2020; Shrestha et al., 2024). Below, we discuss the processes that may have driven these observed color patterns.

### Flower color diversity and pollination systems

Our study revealed that pink, yellow, and white were the predominant flower colors in the campo rupestre, consistent with the most frequent colors found in tropical (Camargo et al., 2019) and temperate communities (Altshuler, 2003; Arnold et al., 2009; Reverté et al., 2016; Bergamo et al., 2018). The prevalence of these colors is linked to the significant role of hymenopteran pollination in the campo rupestre (Carstensen et al., 2014, 2016; Monteiro et al., 2021). Yellow, white, and pink are also the predominant human‐perceived colors of important campo rupestre genera, such as *Vellozia*, *Xyris*, *Chamaecrista*, and *Paepalanthus*.

Plant species with blue‐green and blue flowers were the most frequent bee‐colors, a pattern documented in both tropical and temperate mountainous communities (Arnold et al., 2009, 2010; Bergamo et al., 2018, 2020; Camargo et al., 2019, 2023). However, in the campo rupestre, we found a similar proportion of species with bee‐blue‐green and bee‐blue flowers, whereas temperate regions typically had a predominance of blue‐green over bee‐blue flowers (Arnold et al., 2009, 2010; Bergamo et al., 2018). This difference between neotropical and temperate areas may be attributed to variations in pollinator communities and the relative importance of each pollinator group (Ollerton, 2017).

We found significant relationships between campo rupestre flower color and pollination systems, supporting the pollination syndromes hypothesis (Rosas‐Guerrero et al., 2014; Dellinger 2020). Pollination systems in the campo rupestre have previously been associated with other floral traits, such as flower abundance and size (Carstensen et al., 2014; Monteiro et al., 2021). Our results further reinforce the role of pollinators as agents of flower color selection among angiosperms (Dyer et al., 2012; Shrestha et al., 2013; Camargo et al., 2019). Interestingly, the evidence for flower color defining pollination systems has been mixed (Reverté et al., 2016), which underscores the importance of this trait in the campo rupestre. The open vegetation conditions provide brightness and background contexts that may favor color as a key attractive trait (Bukovac et al., 2017), especially when compared to closed ecosystems such as forests where floral shape, size, and scent may be more important (Machado and Lopes, 2004; Raguso, 2008; Swart et al., 2024), a matter for further investigation.

### Color trait divergence and convergence

Flower color at campo rupestre was more divergent than expected across the environmental gradients. The functional divergence on color traits suggests a stronger role for competition when considering both bee and hummingbird vision (Table 1). Competition for pollinators can lead to divergent color patterns by creating private sensorial niches (Muchhala et al., 2014; Van der Kooi et al., 2016). Since we grouped pollination systems in this analysis, our results may also reflect the co‐occurrence of plants with distinct pollination systems and divergent flower colors in the plots. This divergence might also indicate that environmental filters relevant to flower color are less variable across elevations and vegetation types in the campo rupestre, as we observed no overall color convergence, suggesting a stronger role for biotic interactions.

Nevertheless, when analyzing only bee flowers, we found both divergence and convergence. In this case, closely related yellow bee‐pollinated flowers tend to flower synchronously in the campo rupestre, indicating pollination facilitation (Camargo et al., 2023). Thus, for specific clades and pollination systems, a combination of competition and facilitation may structure flower color assembly (Bergamo et al., 2020), warranting further investigation in the campo rupestre.

We found that the traits driving divergence and clustering patterns increased with elevation, reinforcing the influence of biotic interactions and abiotic factors in regulating the spatial pattern of flower color diversity in the studied community. Contrary to expectations for high elevations (Koski and Ashman, 2015; Bergamo et al., 2018; Gray et al., 2018), traits related to UV‐reflecting flowers (stimulus for bee's UV photoreceptors) increased with elevation. The increase suggests that the solar radiation gradient at higher elevations in this tropical mountain appears insufficient to drive the evolution of enhanced floral UV protection. Additionally, the recently revealed ubiquitous presence of UV‐absorbing compounds in flowers may be sufficient to protect them in our tropical mountain ecosystem and other open vegetation (Narbona et al., 2025). Instead, the increase in UV‐reflecting flowers is more likely related to the importance of short wavelengths such as UV for bee pollination (Shrestha et al., 2013; Camargo et al., 2019; Tunes et al., 2021). It would be important to evaluate floral chemical compounds known to confer UV protection (e.g., flavonoids) to understand the drivers of this increased UV reflectance. Such an increase in UV reflection would also lead to higher brightness (i.e., total reflectance of a flower) at high elevations. However, increased UV reflection should typically lead to lower spectral purity, producing flowers that reflect across multiple wavebands (e.g., white UV‐reflecting flowers). We observed the opposite pattern, likely a result of the increase in UV‐reflecting flowers with relatively higher purity (i.e., yellow UV‐reflecting flowers) at high elevations. This highlights the importance of spectral purity for bee foraging decisions, even at high elevations (Shrestha et al., 2024).

The increased chromatic contrast with the background in hummingbird vision underscores the importance of conspicuousness for hummingbirds along the altitudinal gradient, probably related to the increase in hummingbird vs. bee pollination with elevation in tropical mountains (Dellinger et al., 2023) and in the campo rupestre studied (Monteiro et al., 2021). Such an increase in the richness of hummingbird‐pollinated species may be associated with pressures to enhance floral visibility to ensure successful pollination (Muchhala et al., 2014; Van der Kooi et al., 2016).

### Flower color diversity in the environmental context

Flower color diversity and the importance of pollination systems were similar across vegetation types and only slightly decreased along the altitudinal range. Moreover, while the species phylogenetic structure was related to campo rupestre environmental factors, it had no influence on the diversity of color traits. In the studied mountain sites, phylogenetic and species diversity are linked to elevation and edaphic factors (Mattos et al., 2019, 2021; Loiola et al., 2023). However, while phylogenetic species richness and alpha diversity increase with elevation (Mattos et al., 2019; Loiola et al., 2023), the diversity of color traits was maintained across the campo rupestre landscape. In high mountains of the Alps, Himalayas, and Rocky Mountains, reductions in color diversity with elevation have been observed, following an increased predominance of fly pollination across these gradients (Bergamo et al., 2018; Gray et al., 2018; Shrestha et al., 2014, 2024). Thus, the sustained importance of bee pollination across the environmental gradients in the campo rupestre contrasts with the expected shifts toward the predominance of fly pollination with elevation (Zoller et al., 2023). Nevertheless, an increase in fly pollination with elevation does occur in the campo rupestre (Monteiro et al., 2021), but it may not be sufficient to supersede the importance of bee pollination. The short altitudinal range of campo rupestre maintains diverse abiotic conditions. Even though some vegetation types predominate over others depending on the site, all types are represented along the altitudinal gradient. This creates a mosaic of soil, humidity, and background conditions that favor the maintenance of flower color diversity (Dalrymple et al., 2020).

There were notable exceptions to this stability in flower color diversity, which we linked to specific biotic and abiotic factors. We observed a clear decrease in flower color diversity with elevation in sandy grasslands. The high occurrence of yellow UV‐reflecting flowers in sandy grasslands may have contributed to this, as yellow UV‐reflecting flowers possibly enhances detection against sandy grasslands specific background (Bukovac et al., 2017; Martins et al., 2021a). Conversely, flower color diversity slightly increased with elevation in wet grasslands. Wet grasslands maintain relatively high humidity for longer periods of the year at high elevations (Le Stradic et al., 2015). The long, near year‐round, wet conditions, potentially selects for diverse pigmented flower colors that are more associated with high soil moisture than unpigmented white flowers (Mtileni et al., 2024), matching the reduction in white flowers observed in the studied wet grasslands.

We also highlight specific associations between flower color with elevation and vegetation types. We found a high frequency of pink UV‐absorbing flowers, corresponding to bee‐blue flowers, in stony grasslands. Such colors are highly attractive to bees (Lunau et al., 1996) and may contrast particularly well with the stony grassland background. There was a higher frequency of red UV‐absorbing flowers in the rocky outcrops. Hummingbird pollination is often associated with red UV‐absorbing colors (Lunau et al., 2011; Bergamo et al., 2016; Camargo et al., 2019) and is also the predominant pollination system in the campo rupestre rocky outcrops (Monteiro et al., 2021; Lopes et al., 2023). Finally, the frequency of flowers with pollen/anther mimicking structures that are related to bee pollination, also increased with elevation, promoting protection of reproductive parts against UV‐light (Lunau et al., 2017, 2024).

## CONCLUSIONS

To our knowledge, our study represents the first quantitative evaluation of flower color and color signal distribution across a tropical mountain gradient, explicitly testing the influence of biotic interactions, abiotic variables, phylogeny, and species composition. We found evidence supporting an association between pollination systems and flower color, along with a predominance of color traits related to bee pollination, reinforcing the importance of the bee pollination system across the campo rupestre landscape (Guerra et al., 2016; Monteiro et al., 2021). Furthermore, the diversity of flower color traits is maintained across the vegetation mosaic and elevation in the campo rupestre. The detected functional stability contrasts sharply with reductions in color diversity observed in temperate high mountains with increasing elevation (Bergamo et al., 2018; Gray et al., 2018; Shrestha et al., 2024). Our results suggest that flower color diversity is maintained across environmental gradients when pollination systems are not limited by the altitudinal gradient and abiotic conditions remain variable but not extreme, a characteristic of a tropical snow‐free mountain system.

## AUTHOR CONTRIBUTIONS

M.G.G.C. and L.P.C.M. conceived the study and collected data in the field. M.G.G.C., B.L.M. and L.P.C.M. organized and curated the data set. M.G.G.C. and P.J.B. conducted statistical analyses. M.G.G.C., M.A., and L.P.C.M. wrote the first draft of the manuscript. All authors have critically contributed to the final version of the manuscript.

## Supporting information


**Appendix S1.** Supplemental tables and figures.
**Table S1.** Number of plots, botanical families, genera, species, and species per pollination system.
**Table S2.** Number of species and frequencies and the percentage of species and records per pollination system of each flower color.
**Table S3.** Blomberg's *K* values for quantitative color variables.
**Figure S1.** Location of the Espinhaço Mountain Range in South America.
**Figure S2.** Correspondence analyses between the frequency of pollination systems and the frequency of bee color.
**Figure S3.** Percentage of pairs of flower color loci by classes of distance in the bee color space and the bird color space.

## Data Availability

All supporting data are available in the Zenodo repository at https://doi.org/10.5281/zenodo.17237031 (Camargo et al., 2025).
