# Supplementary material for: A mosaic of colors: The influence of biotic and abiotic factors shaping flower color diversity across a tropical mountain ecosystem
Source: Am J Bot. 2026 Jan 9;113(1):e70147. doi: 10.1002/ajb2.70147 (PMC12816440; doi:10.1002/ajb2.70147)
Supplement: Supplementary file 1 — Appendix S1. Supplemental tables and figures. Table S1. Number of plots, botanical families, genera, species, and species per pollination system. Table S2. Number of species and frequencies and the percentage of species and records per pollination system of each flower color. Table S3. Blomberg's K values for quantitative color variables. Figure S1. Location of the Espinhaço Mountain Range in South America. Figure S2. Correspondence analyses between the frequency of pollination systems and the frequency of bee color. Figure S3. Percentage of pairs of flower color loci by classes of distance in the bee color space and the bird color space. [file AJB2-113-e70147-s001.docx]

**Appendix S1.** Supporting tables and figures.

**Table S1.** Number of plots, botanical families, genera, species, and species per pollination system and frequency (number of records) of pollination system for plant species sampled in the studied campo rupestre, and with flower reflectance data, for all studied sites (General), for each altitude and for each vegetation type. Pollination systems were classified as bee, hummingbird (hum), and other systems, which included diverse insects (div), fly, moth (mot), wasp (was), butterfly (but), and bat.

| Sites | Plots | Families | Genera | Species | Number of species | | | | | | | |  | Number of records | | | | | | | | |
| --- | --- | --- | --- | --- | --- | --- | --- | --- | --- | --- | --- | --- | --- | --- | --- | --- | --- | --- | --- | --- | --- | --- |
|  |  |  |  |  | bee | hum | Other systems | | | | | |  | bee | hum | Other systems | | | | | | Total |
|  |  |  |  |  |  |  | div | mot | fly | was | but | bat |  |  |  | div | mot | fly | was | but | bat |  |
| General | 180 | 46 | 106 | 179 | 134 | 14 | 17 | 3 | 6 | 3 | 1 | 1 |  | 845 | 67 | 99 | 5 | 47 | 13 | 2 | 1 | 1079 |
| Rio Cipó (824 m) | 36 | 25 | 49 | 57 | 44 | 5 | 5 | 1 | 0 | 1 | 0 | 1 |  | 96 | 12 | 30 | 3 | 0 | 1 | 0 | 1 | 143 |
| Cedro (1101 m) | 36 | 27 | 43 | 61 | 47 | 5 | 5 | 1 | 1 | 2 | 0 | 0 |  | 126 | 15 | 6 | 1 | 1 | 6 | 0 | 0 | 155 |
| Elefante (1255 m) | 36 | 31 | 57 | 86 | 66 | 6 | 8 | 1 | 3 | 2 | 0 | 0 |  | 239 | 13 | 14 | 1 | 3 | 4 | 0 | 0 | 274 |
| Quadrante 16 (1303 m) | 36 | 30 | 49 | 71 | 57 | 5 | 4 | 0 | 4 | 1 | 0 | 0 |  | 191 | 22 | 11 | 0 | 6 | 1 | 0 | 0 | 231 |
| Alto Palácio (1420 m) | 36 | 19 | 36 | 56 | 40 | 2 | 6 | 0 | 6 | 1 | 1 | 0 |  | 193 | 5 | 38 | 0 | 37 | 1 | 2 | 0 | 276 |
| Cerrado | 38 | 23 | 46 | 58 | 46 | 5 | 5 | 1 | 0 | 1 | 0 | 0 |  | 106 | 10 | 28 | 3 | 0 | 2 | 0 | 0 | 149 |
| Rocky outcrop | 38 | 34 | 70 | 103 | 77 | 10 | 10 | 1 | 2 | 2 | 0 | 1 |  | 187 | 30 | 13 | 1 | 4 | 9 | 0 | 1 | 245 |
| Sandy grassland | 55 | 27 | 52 | 83 | 65 | 5 | 7 | 0 | 6 | 0 | 0 | 0 |  | 293 | 16 | 41 | 0 | 33 | 0 | 0 | 0 | 383 |
| Stony grassland | 39 | 26 | 49 | 76 | 60 | 3 | 5 | 1 | 5 | 1 | 1 | 0 |  | 240 | 11 | 13 | 1 | 8 | 1 | 2 | 0 | 276 |
| Wet grassland | 10 | 15 | 18 | 23 | 16 | 0 | 4 | 0 | 2 | 1 | 0 | 0 |  | 19 | 0 | 4 | 0 | 2 | 1 | 0 | 0 | 26 |

**Table S2.** Number of species (*N* spp) and frequencies (number of records per plots; *N* records) and the percentage of species and records per pollination system of each flower color based on the human color vision associated to the UV-reflectance (indicated by “uv” after the color category), bee-color categories and according to the presence of color pattern, UV-pattern, pollen/anther mimic structures and other floral guides. We sampled a total of 179 plant species with 1079 records in the 180 plots (1 m^2^) established in the studied campo rupestre (Serra do Cipó, Minas Gerais State, Brazil). Pollination systems were classified as bee (134 species with 845 records), hummingbird (hum; 14 species with 67 records), and other systems (other, 31 species with 167 records). The category “other” included diverse insects, fly, moth, wasp, butterfly, and bat.

| Variable | *N* spp | % of species per pollination system | | |  | *N* records | % of records per pollination system | | |
| --- | --- | --- | --- | --- | --- | --- | --- | --- | --- |
|  |  | bee | hum | other |  |  | bee | hum | other |
| Pink | 50 | 34.3 | 14.3 | 6.5 |  | 284 | 32.2 | 7.5 | 4.2 |
| White | 33 | 17.9 | 21.4 | 19.4 |  | 244 | 21.3 | 46.3 | 19.8 |
| Yellow | 14 | 9.0 | 7.1 | 3.2 |  | 59 | 6.5 | 3.0 | 1.2 |
| Whitish | 26 | 6.7 | 0.0 | 54.8 |  | 127 | 4.4 | 0.0 | 53.9 |
| Red | 6 | 1.5 | 28.6 | 0.0 |  | 33 | 1.4 | 31.3 | 0.0 |
| Green | 1 | 0.0 | 0.0 | 3.2 |  | 1 | 0.0 | 0.0 | 0.6 |
| Pink-uv | 11 | 7.5 | 7.1 | 0.0 |  | 42 | 4.7 | 3.0 | 0.0 |
| White-uv | 2 | 0.7 | 0.0 | 3.2 |  | 19 | 0.1 | 0.0 | 10.8 |
| Yellow-uv | 30 | 20.9 | 14.3 | 0.0 |  | 244 | 28.3 | 7.5 | 0.0 |
| Whitish-uv | 3 | 0.7 | 7.1 | 3.2 |  | 10 | 0.6 | 1.5 | 2.4 |
| Red-uv | 2 | 0.7 | 0.0 | 3.2 |  | 9 | 0.5 | 0.0 | 3.0 |
| Green-uv | 1 | 0.0 | 0.0 | 3.2 |  | 7 | 0.0 | 0.0 | 4.2 |
|  |  |  |  |  |  |  |  |  |  |
| Bee-bluegreen | 60 | 27.6 | 21.4 | 64.5 |  | 374 | 28.5 | 46.3 | 61.1 |
| Bee-blue | 52 | 34.3 | 28.6 | 6.5 |  | 291 | 32.3 | 16.4 | 4.2 |
| Bee-uvgreen | 30 | 20.9 | 7.1 | 3.2 |  | 219 | 24.4 | 1.5 | 7.2 |
| Bee-green | 20 | 9.7 | 21.4 | 12.9 |  | 82 | 6.9 | 13.4 | 9.0 |
| Bee-uvblue | 15 | 6.0 | 21.4 | 12.9 |  | 74 | 3.3 | 22.4 | 18.6 |
| Bee-uv | 2 | 1.5 | 0.0 | 0.0 |  | 39 | 4.6 | 0.0 | 0.0 |
|  |  |  |  |  |  |  |  |  |  |
| Pattern | 118 | 78.4 | 57.1 | 16.1 |  | 753 | 83.7 | 34.3 | 13.8 |
| UV pattern | 42 | 29.1 | 14.3 | 3.2 |  | 281 | 32.4 | 3.0 | 3.0 |
| Mimicry | 58 | 41.8 | 7.1 | 3.2 |  | 362 | 41.4 | 1.5 | 6.6 |
| Other guides | 23 | 16.4 | 7.1 | 0.0 |  | 102 | 11.8 | 3.0 | 0.0 |

**Table S3.** Blomberg’s *K* values for quantitative color variables of flowers from campo rupestre species (Serra do Cipó, Minas Gerais State, Brazil). PICobs: phylogenetic independent contrasts observed; PICrnd: phylogenetic independent contrasts randomized. Significant values are in bold (*P* < 0.05). Tested variables calculated according to the bee’s visual system: UV, blue and green photoreceptor stimulus, chromatic and green contrasts, spectral purity and color intensity; tested variables calculated according to the bird’s visual system: chromatic and achromatic contrasts and UV and red photoreceptor stimulus.

| **Visual system** | *K* | PICobs | PICrnd | *P* |
| --- | --- | --- | --- | --- |
| **Bee** |  |  |  |  |
| UV stimulus | 0.11 | 0.001 | 0.001 | 0.10 |
| Blue stimulus | 0.15 | 0.002 | 0.003 | **0.01** |
| Green stimulus | 0.06 | 0.002 | 0.002 | 0.68 |
| Chromatic contrast | 0.20 | 0.000 | 0.000 | **0.00** |
| Color intensity | 0.07 | 0.012 | 0.012 | 0.53 |
| Spectral purity | 0.12 | 0.001 | 0.002 | 0.11 |
| Green contrast | 0.11 | 0.001 | 0.001 | 0.08 |
|  |  |  |  |  |
| **Bird** |  |  |  |  |
| Chromatic contrast | 0.11 | 0.537 | 0.769 | 0.15 |
| Achromatic contrast | 0.12 | 1.319 | 2.195 | 0.05 |
| UV stimulus | 0.25 | 0.000 | 0.000 | **0.00** |
| Red stimulus | 0.06 | 0.001 | 0.001 | 0.75 |

**
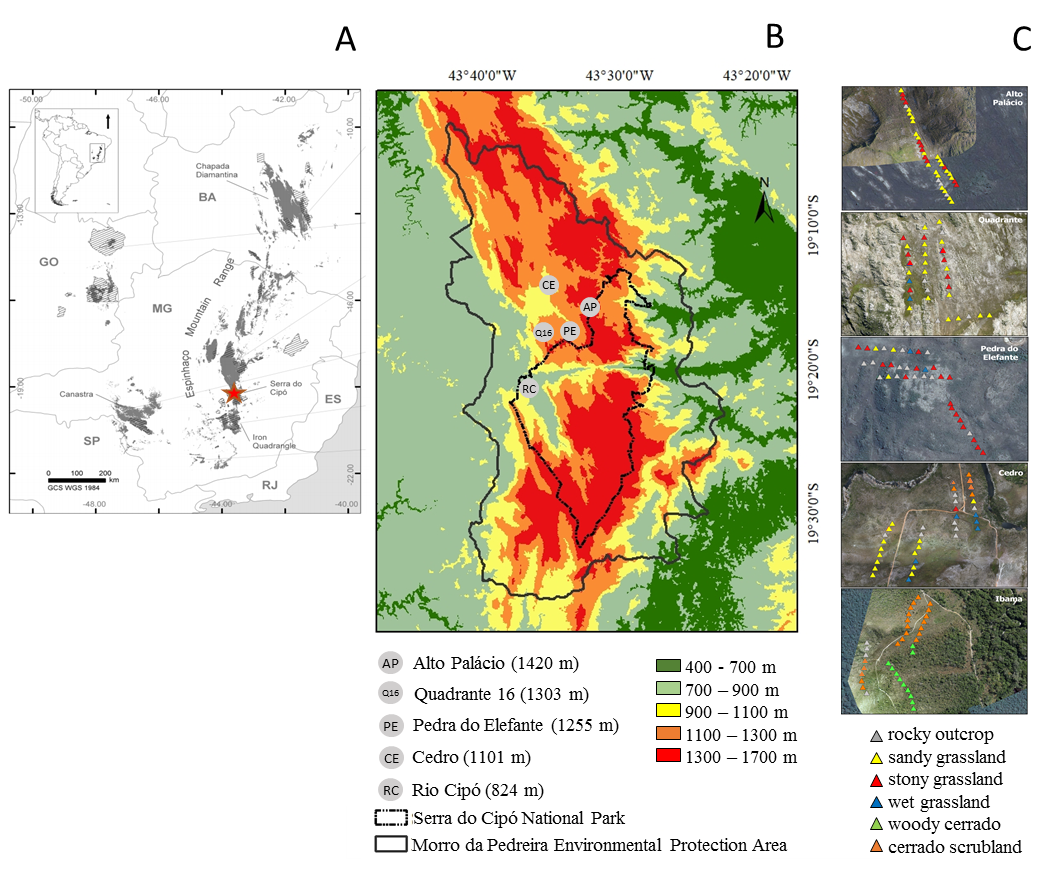
**

**Figure S1.** Location of (A) the Espinhaço Mountain Range in South America and detailed of the range in Bahia and Minas Gerais States in Brazil, with the Serra do Cipó study site indicated by a red star (source: Silveira et al., 2016). (B) Detail of the limits of the Serra do Cipó National Park and its buffer zone, the Morro da Pedreira Environmental Protection Area, showing the five collection sites position and altitude (adapted from Alvarado et al., 2017). (C) Detail of the five collection sites showing the transects and plots position and respective vegetation types (source of images: Mattos et al. (2019) and e-phenology Fapesp-Microsoft project (#2013/50155–0).


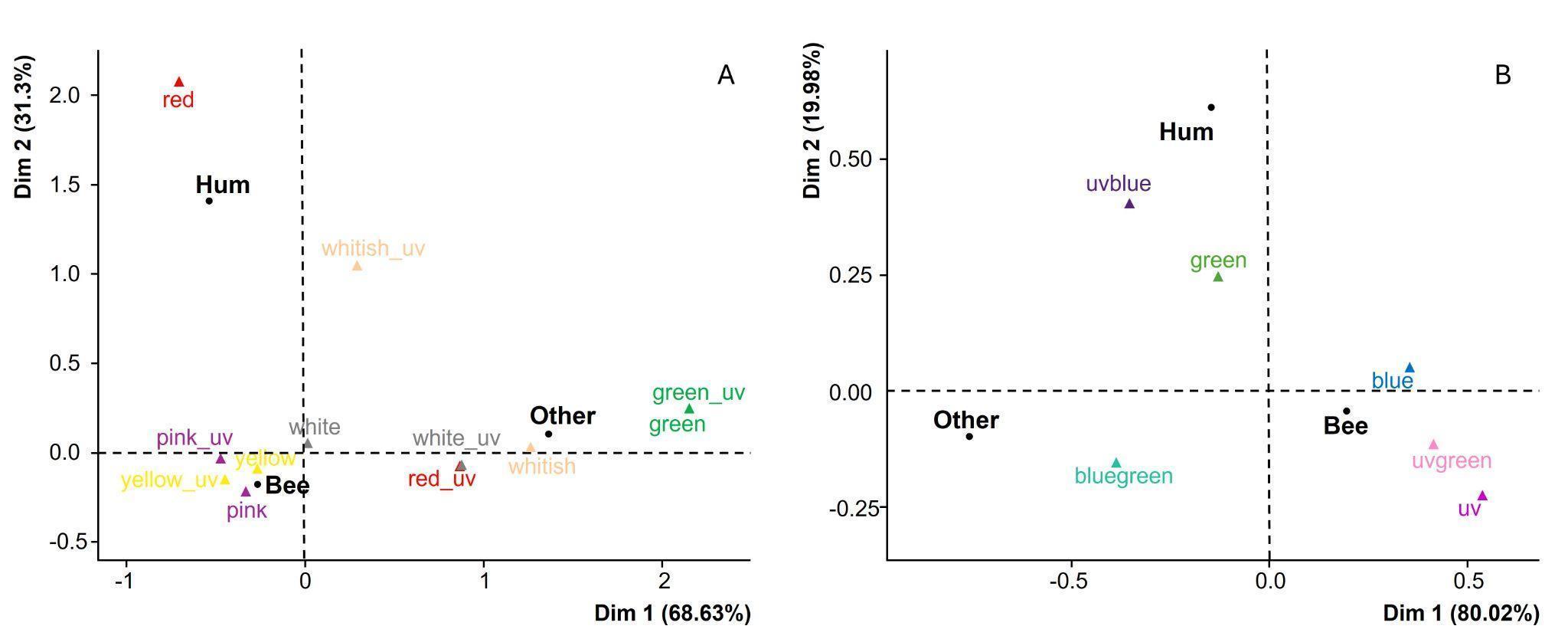


**Figure S2.** Correspondence analyses between the frequency of pollination systems (black) and the frequency of bee-color (red) of flowers sampled in the studied campo rupestre (Serra do Cipó, Minas Gerais State, Brazil). (A) Human colors associated with UV reflectance (“_uv” indicates UV-reflecting colors). (B) Bee-color categories were calculated in the bee-hexagon (Chittka et al. 1994).


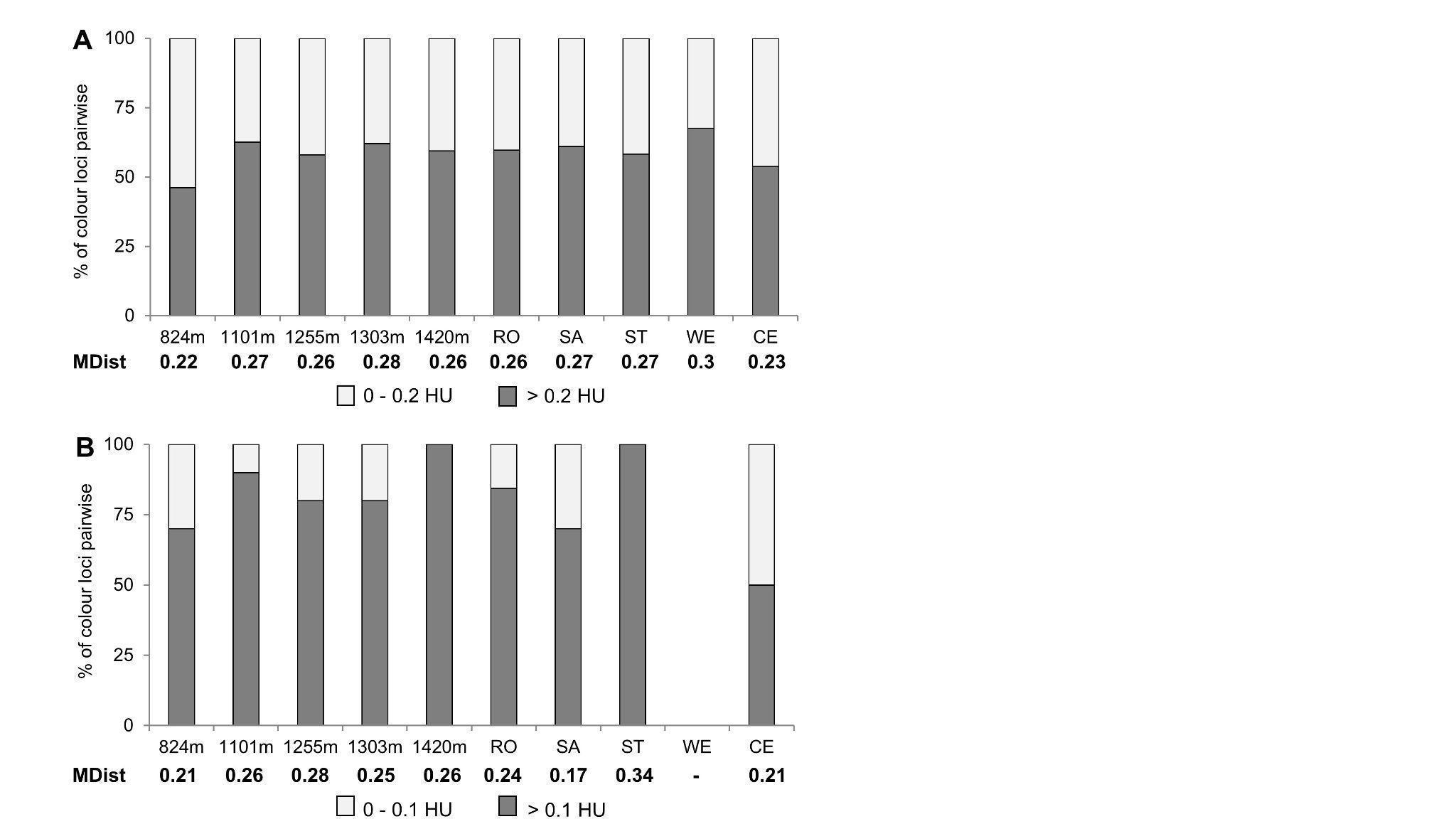


**Figure S3.** Percentage of pairs of flowers’ color loci by classes of distance in the (A) bee color space (bee-hexagon) for bee-pollinated flowers, and (B) in the bird color space (tetrahedron) for hummingbird-pollinated flowers in each altitude and vegetation type. Bees can discriminate among two color loci more than 0.2 hexagon units (HU) distant from each other, and this value is around 0.1 just noticeable differences (JND) for birds. The mean distance (MDist) among flowers’ color loci and the total number of color loci pairs (*N*) of each community is also given . (CE) cerrado; (RO) rocky outcrop; (SA) sandy grassland; (ST) stony grassland; wet grassland (WE).

**References**

Chittka, L., A. Shmida, N. Troje, and R. Menzel. 1994. Ultraviolet as a component of flower reflections, and the colour perception of Hymenoptera. *Vision Research* 34: 1489-1508.

Mattos, J. S., L. P. C. Morellato, M. G. G. Camargo, and M. A. Batalha. 2019. Plant phylogenetic diversity of tropical mountaintop rocky grasslands: local and regional constraints. *Plant Ecology* 220: 1119–1129.
